# Conversation with Future Clinical Cytogeneticists: The New Frontiers

**DOI:** 10.3390/genes17020232

**Published:** 2026-02-12

**Authors:** Jing Christine Ye, Rishi Chowdhury, Henry H. Heng

**Affiliations:** 1 Division of Cancer Medicine, Department of Lymphoma/Myeloma, University of Texas MD Anderson Cancer Center, Houston, TX 77030, USA; jcye@mdanderson.org; 2 Center for Molecular Medicine and Genetics, Wayne State University School of Medicine, Detroit, MI 48201, USA; 3 Department of Pathology, Wayne State University School of Medicine, Detroit, MI 48201, USA

**Keywords:** Genome Architecture Theory, cancer drug resistance, non-clonal chromosome aberrations or NCCAs, information management, karyotype coding, system level inheritance, two-phased evolution

## Abstract

The post-genomic era has transformed medical genetics, raising renewed debate over the role of medical cytogenetics in clinical practice. High-throughput sequencing and chromosomal microarray technologies now dominate cancer diagnostics, prenatal testing, and rare disease evaluation by enabling rapid detection of gene-level variation, often leading to the perception that cytogenetics is obsolete. However, this view overlooks the unique and complementary strengths of cytogenetic analysis. Although the relationship between cytogenetics and current NGS technologies can be compared to that between forests and trees versus leaves—both of which are necessary for clinical diagnosis—cytogenetic methods uniquely enable direct in situ visualization of chromosomes, allowing detection of large-scale structural and numerical genome alterations at the level of individual cells and cell populations. These system-level features that are frequently invisible or difficult to interpret using sequencing-based approaches alone yet are critical in disease contexts where genome architecture itself carries biological and clinical significance beyond individual genes. This article, therefore, advances a new perspective based on Genome Architecture Theory: that karyotype-level information organizes gene-level function and that many previous gene-centric genetic concepts require reexamination within a unified framework of clinical genomics. Rather than being replaced, cytogenetics is increasingly integrated with sequencing within a unified framework of clinical genomics that combines high-resolution molecular detail with system-level insight into genome organization. Reassessing the role of cytogenetics, therefore, has important implications for medical education, diagnostic strategy, and healthcare policy, as cytogenetics provides the appropriate platform for understanding system-level inheritance through karyotype coding and for advancing molecular medicine from a genome systems perspective.

## 1. Introduction

The gene era has transformed our understanding of human disease by focusing on molecular profiles and reshaping how clinical genetics is practiced. A major expectation of this era was that once the entire human genome could be sequenced, the mechanisms underlying most—if not all—diseases would be revealed, leading naturally to effective cures [1,2,3]. Yet, more than 20 years after the completion of the Human Genome Project, this promise has not been realized [4,5,6,7,8]. The gap between molecular data and clinical outcomes raises an essential question: what should be the future direction of clinical genetics [9,10]?

Many researchers remain hopeful that simply collecting more molecular data—especially with increasingly powerful computational tools—will eventually fulfill the original promise of the gene era. Yet others, although still a minority, have begun to seek explanations at the epigenetic level or, more importantly, at higher system levels, recognizing that disease mechanisms cannot be fully captured by gene-by-gene analyses alone [11,12,13,14,15]. As biology enters a new era of integrative, multi-omics, and big-data research, the limitations of a purely molecular focus are becoming increasingly apparent. How can we develop experimental platforms that integrate environmental impacts on genetic profiles [4], address missing heritability and fuzzy-inheritance-based heterogeneity [16,17,18,19], interpret the large amount of genetic variation that lacks clear disease-phenotype correlations, and resolve conflicts between gene-level data and chromosomal-level information [20,21,22,23]?

This shift has naturally revived discussions about the importance of clinical cytogenetics—a field that was overshadowed during the gene-centric decades [4,9,24,25,26,27,28]. The renewed interest is not accidental; it reflects a deeper logic concerning the relationship between genes and chromosomes and the multi-scale organization of biological inheritance.

For example, chromosomes serve not only as the physical vehicles of genes but also as the immediate environment within which genes interact. The karyotype represents the highest level of genetic and genomic organization, providing the system-level architecture that determines how genes are arranged and how their interactions are coordinated [4,24]. Chromosomal abnormalities are associated with a broad spectrum of diseases, and their frequencies far exceed those of most single-gene disorders. Comparatively, any chromosomal change affects hundreds of genes, if not more. Furthermore, across species, distinct karyotypes correspond to distinct system identities, underscoring the system-level role of genome organization.

This is why, in recent years, system-level genomic information, particularly karyotype organization, has emerged as essential for understanding genomics and disease evolution [4,9], predicting clinical trajectories, and explaining why molecular profiles alone often fail to yield actionable insights. From this broader perspective, cytogenetics is not an outdated discipline; rather, it provides the foundational organizational framework of the genome. It is the level at which molecular information—especially gene–environment interactions—is integrated, regulated, and transformed into biological and disease phenotypes. Consequently, cytogenetics and cytogenomics are poised to play a central role in the future of genomic medicine [10,29,30,31,32].

The following questions briefly summarize several representative issues and offer answers based on Genome Architecture Theory [4,29,33,34,35]. We hope that these new perspectives will help advance the modernization and refinement of clinical cytogenetics and cytogenomics.

## 2. Why Do We Need Such Discussions?

Molecular medicine has long promised to revolutionize medical diagnosis and treatment. As a subfield of medical genetics, clinical cytogenetics has been deeply influenced by the conceptual and technological advances of molecular genetics. The cloning of a large number of disease-associated genes and the widespread availability of high-throughput sequencing have made it possible to efficiently obtain mutation and copy number variation (CNV) profiles with high precision and automation [36,37]. These approaches appear more reliable and cost-effective due to advances in automated workflows and user-friendly tools for computational data analysis [38].

In contrast, classical cytogenetics requires extensive training, experience-based interpretation, and time-consuming laboratory procedures. Consequently, a growing number of studies and commentaries have suggested that cytogenetics has already experienced its “golden age” and is now being superseded by sequencing-based platforms. As cytogenetics has begun to fall out of favor, it has become increasingly challenging to sustain progress and attract new generations of researchers to advance the field. The very fact that the field must now defend its importance reflects the depth of these challenges. Although discussions about revitalizing cytogenetics have increased, most focus primarily on technological narratives; however, this emphasis risks overlooking the deeper conceptual and system-level significance of cytogenetics [39,40,41,42,43,44,45,46,47,48].

Unexpectedly, however, molecular cytogenetics and cytogenomics are quietly making a comeback—both conceptually and experimentally. This revival has been driven by new discoveries showing that cancer macroevolution is defined by karyotype reorganization rather than by gene mutation [49,50] and that cytogenetic heterogeneity underlies key mechanisms such as drug resistance [51], the formation of polyploid giant cancer cells [52,53,54,55,56,57,58,59,60,61,62,63,64,65] and even cell death as an effective means of generating new cancer systems [66,67,68,69].

These findings also help explain why even the most powerful DNA sequencing technologies remain limited for clinical prediction and why karyotype and gene represent distinct yet complementary forms of inheritance and biological information. Furthermore, this new perspective reveals the conflict and complex relationship between specific molecular pathways and system-level behaviors. Biological systems respond to stress in scale-dependent and non-linear ways, where emergent transitions—rather than simple accumulative mutations—govern adaptive outcomes across different evolutionary phases.

Taken together, these insights form the foundation of a new genome- and information-based genomic and evolutionary framework—known as the Genome Architecture Theory of evolution—which may redefine the future direction of genomics, including clinical cytogenetics [4]. This emerging framework offers an excellent opportunity to engage in a timely dialogue about the new perspectives shaping cytogenetics and cytogenomics in the twenty-first century [9]. Specifically, the key message is that cytogenetics and cytogenomics will not be replaced by sequencing technologies; rather, they will become essential disciplines that organize, interpret, and contextualize sequencing data—making genomic information truly useful for future medicine.

## 3. Cytogenetic Discoveries Have Been Widely Misunderstood and Insufficiently Appreciated

Even before molecular genetics became dominant, the conceptual importance of cytogenetics was often misunderstood and undervalued. In evolutionary biology, for instance, cytogeneticists had long emphasized the significance of karyotype organization in speciation [70]—how chromosomal rearrangements and structural variations could define new species boundaries. Yet, this system-level view was largely ignored by mainstream evolutionary biologists, who focused instead on gene-level mutations as the main driver of evolutionary change. The fascination with molecular resolution fostered a reductionist mindset, as if understanding smaller components automatically meant understanding the whole system. However, if this reasoning were valid, one could just as well argue that studying atoms would reveal all of biology. True understanding requires acknowledging that higher-order organization, such as the karyotype structure, encodes system-level information that cannot be reduced to the sum of its molecular parts.

In the field of genetics, Barbara McClintock, one of the most remarkable cytogeneticists in history, revered the genome as an ultimate genetic control system. Her decades-long series of elegant experiments was breathtaking in its insight, yet many contemporaries dismissed or ignored her discoveries—often not because they could refute her data, but because her conceptual framework was far ahead of its time and too difficult for others to grasp within the prevailing paradigm. For example, in her series of genome-manipulating experiments using hybridization to induce genome reorganization, McClintock created what has been described as a “genetic earthquake,” linking genome restructuring to new patterns of inheritance by activating transposable elements and reshuffling the genome [71,72]. Although her discovery of “jumping genes” eventually earned her the Nobel Prize, McClintock’s deeper insight—the dynamic genome as an information control system—remains underappreciated by most researchers today [4,73].

From the perspective of the Genome Architecture Theory, however, we can now fully appreciate her vision: she was describing inheritance at the system level, in contrast to the Mendelian focus on the mechanisms of individual genetic parts. When McClintock urged scientists to “focus on the genome,” she was, in essence, pointing to the karyotype as the ultimate organizer of genetic information—a concept only now finding its true resonance in modern cytogenetics [74]. What a visionary she was.

When studying a model of spontaneous cellular immortalization, we traced karyotype evolution in a “watching evolution in action” experiment. By comparing phenotypes (such as growth behavior, senescence, cell death, population emergence, and replacement) with genotypes—or more precisely, *genomitypes*—we made several surprising observations:

- The cloning of DNA sequences and the cloning of karyotypes are fundamentally different processes, suggesting that distinct forms of inheritance are involved.
- Cancer evolution follows a two-phased process: a macro-evolutionary phase, driven by karyotype reorganization and characterized by a rapid and discontinuous process coupled with massive cell death; and a micro-evolutionary phase, driven by gene and epigenetic alterations that promote population growth and characterized by a gradual process coupled with stepwise competition.
- The phase transition is co-mapped with the highest level of genomic heterogeneity, among which genome chaos represents an extreme form. Environmental crises, such as massive cell death, act as triggers for genome-chaos–mediated phase transitions.

## 4. Extensive Karyotypic Heterogeneity Is Essential for Each Major Evolutionary Phase Transition in Cancer, Including Transformation, Metastasis, and Drug Resistance

In addition to the immortalization process, such karyotype-mediated macro–microevolutionary transitions are observed across diverse cancer evolutionary events, including transformation, metastasis, and drug resistance, even though their underlying molecular mechanisms can differ substantially. In this sense, cancer formation can be viewed as an information-generating and amplifying process. During crisis episodes, when environmental stressors trigger the process, genome chaos acts as a central platform for creating new system-level information, followed by the stochastic recruitment of gene mutations or epigenetic alterations that provide growth advantages and lead to clonal dominance. Through repeated cycles of this two-phased evolution, cancer evolves toward clinically significant states.

Compared with McClintock’s observations, our studies also focused on the macroevolutionary process, illustrated through the interactions between phenotype and genomitype. Furthermore, we clarified the mechanism of two-phased evolution and explicitly linked it to two distinct types of inheritance. We also recognized that the evolutionary process, by and large, functions to constrain the genome, while speciation is primarily achieved through genome reorganization.

Initially, we faced significant challenges publishing our molecular cytogenetic data, partly due to the lack of experts familiar with cytogenetics, evolution, and cancer biology, and partly because reviewers tended to favor gene-centered research. Chaotic genomes were often dismissed as insignificant dead ends, and the concept of genome chaos was considered irrelevant to evolution, since only small, incremental alterations were thought to drive cancer progression. This was despite clear evidence that chromosomal changes—not incremental alterations—are overwhelmingly dominant. Many biologists also assumed that biological systems are so elegantly organized that they could not possibly operate through such chaotic mechanisms. Later, with the accumulation of large-scale cancer genome sequencing data, it became clear that genome-level alterations are pervasive across all types of cancers. The chaotic genomes originally identified through cytogenetic research were finally confirmed by sequencing data—though often described under different names, such as chromothripsis, chromoplexy, and chromoanagenesis, among others [75,76,77,78,79,80]. It is also possible that within a chaotic genome, DNA-level instability, including microsatellite instability, can increase when higher-order genome constraints are reduced. It is important to emphasize that while sequencing technology validated the existence of chaotic genomes, confirming that they are real and not artifacts, it did not *discover* them. These findings stem from the painstaking and visionary observations made by cytogeneticists.

Another idea is that the phase transition is so drastic that it requires changes in information at the level of the entire system, enabling the effective balancing of different pathways rather than relying on localized genetic changes alone. Coordinated changes across multiple components can generate large-scale novelty.

## 5. Why Karyotype Analysis Is of Ultimate Importance: The Concept of Karyotype Coding

Large-scale sequencing projects have unexpectedly revealed the limitations of gene-centric approaches in explaining the mechanisms underlying the most common and complex diseases, where numerous genetic and non-genetic factors interact. Early expectations that cancer could be explained by a small number of driver genes have proven incorrect [81]; instead, thousands of gene mutations have now been identified across different tumor types [82,83,84]. To address such complexity, systems biology emerged with the promise of studying biological phenomena at the system level rather than focusing on individual components. However, despite this promise, unifying principles of systems biology have yet to be firmly established. In particular, simply accumulating more data does not equate to systemic thinking.

A fundamental unresolved question remains: how does genetics define system-level inheritance if it cannot be adequately explained by individual genes or even combinations of genes? In addition, what is the genomic basis of non-typical dominant or recessive traits, including incomplete dominance, codominance, quantitative traits, and multiple allelism? These phenomena reflect the limited expressive capacity of individual genes, indicating that gene-centric explanations need to be revised within the context of diverse modes of inheritance. Furthermore, genome-wide association studies (GWAS) have consistently shown that many disease-associated loci reside in non-coding regions of the genome. This observation suggests that profiling gene mutations alone is insufficient to resolve the inheritance of complex diseases. Instead, it opens the door to alternative forms of inheritance, including karyotype-defined, system-level inheritance—an aspect long overlooked by gene-centric, deterministic views of genetics.

The answer, once again, emerged from cytogenetic studies of cancer evolution [50]. By comparing the roles of gene mutations and karyotype alterations during cancer evolution, it became evident that the concept of a clone can be defined from both a gene-centric and a karyotype-focused perspective, with each narrative leading to fundamentally different interpretations. For example, identical DNA sequences can exist within different karyotypic contexts, demonstrating that DNA clones and karyotype clones represent distinct biological realities.

**Figure 1:**
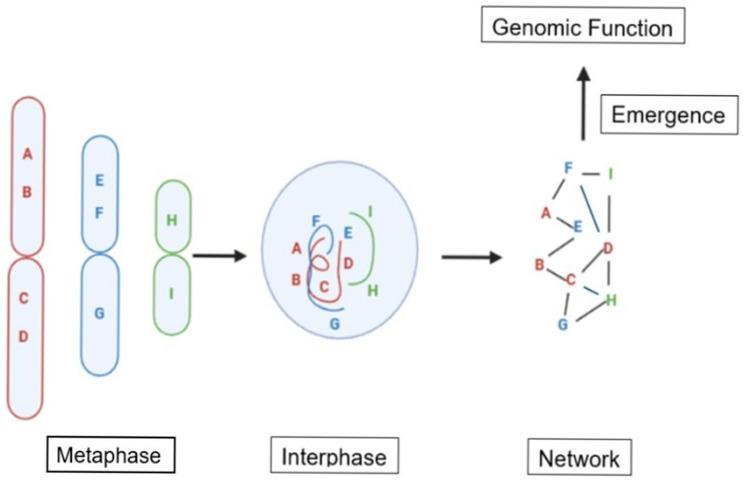
Illustrates the proposed concept of karyotype coding and its relationship to chromatin domains, network structure, various regulatory mechanisms, including epigenetic impacts and modifications below the gene level, which contribute to genomic function. The order of genes is schematically labeled on three chromosomes (A, B, C, D, E, F, G, H and I). In the interphase nucleus, these three chromosomes exist as distinct chromatin domains (shown in red, blue, and green). Because each chromatin domain occupies a unique topological position within the nucleus, it establishes specific patterns of inter-domain interaction, thereby constraining active gene interactions through chromatin domain organization. Due to necessary simplification, only chromosomes, chromatin domains, and network structures are illustrated to convey the central point, even though multiple levels of information modification are involved in any given genomic function, including subcellular environmental constraints.

These observations indicate that genes and karyotypes correspond to different modes of inheritance: part-level inheritance and system-level inheritance, respectively. While part-level inheritance is well understood to be encoded by the genetic code, an equivalent framework for system-level inheritance has long been missing. It was therefore proposed that the order, arrangement, and contextual organization of genes and regulatory elements along chromosomes constitute a higher-order code—termed *karyotype coding*. In this view, karyotype coding defines system-level network architecture by constraining and shaping gene interactions, thereby specifying biological organization beyond individual gene function. A simple illustrative diagram is presented in Figure 1.

These constraints are reflected in the spatial “address” of genes within the nucleus and collectively define the structure of gene interaction networks. Through dynamic interactions with the cellular environment, these networks give rise to emergent genomic functions. In this framework, genome organization—reflected in broader syntenic relationships rather than individual gene identity alone—plays a central role in shaping system-level interactive behavior among genomic components.

As previously discussed [4,24,29,33,85], multiple lines of evidence support the importance of gene clusters and syntenic organization in both development and speciation. These include: (i) the critical role of synteny in regulating developmental programs and key biological processes, such as information management and energy flow; (ii) the strong association between aneuploidy, including experimentally transferred small chromosomal fragments, and genome-wide expression changes that extend beyond simple gene dosage effects, as further supported by transcriptomic analyses; (iii) the recognition of most cancers as newly emergent cellular systems, or quasi-cellular species, characterized by altered karyotypes and drastic phenotypes; (iv) the problem of missing heritability can be explained, at least in part, by karyotype coding [16]; and (v) the observation that, despite sharing highly similar gene content, mammalian species differ substantially in chromosomal organization.

We further predict that changes in the genomic “code” at the karyotype level generate new, system-wide gene expression packages, within which the functional roles of individual genes may be altered. Moreover, different species, and pathological cellular systems such as cancer, exhibit distinct karyotype coding, reflecting the emergence of new biological systems. This framework further suggests that many diseases are associated with alterations in system-level inheritance rather than changes in individual genes alone.

Notably, although karyotype coding represents a concept distinct from and hierarchically above genetic coding—and therefore raises more questions than it immediately answers—this hypothesis provides a coherent framework for understanding multiple levels of biological information, particularly their hierarchical organization with temporal and spatial specificity. Encouragingly, the concept of karyotype coding has recently gained support from several related models and conceptual frameworks. These studies suggest that syntenic relationships and large-scale DNA organization can have functional significance and that unique centromeric sequences may represent higher-order coding beyond individual genes [33,86]. If correct, system-level coding offers a direct explanation for why karyotype analysis remains essential for understanding development, evolution, and disease. For a more detailed discussion, see Chapter 4 of the *Genome Chaos*, second edition [4].

A frequently raised question concerning karyotype coding is how it differs from genetic coding. Genetic coding is digital in nature, with universal specificity shared across species. In contrast, karyotype coding does not appear strictly digital: each species possesses a unique karyotype, there is no simple one-to-one correspondence between karyotype changes and phenotypic outcomes, and not all altered karyotypes immediately produce distinct phenotypes.

This apparent discrepancy becomes understandable when genetic coding is viewed as specifying parts or components, whereas karyotype coding defines system-level design. The digital precision and universality of the genetic code are appropriate for encoding conserved molecular components, while system design—like architecture—can vary widely even when built from identical parts. As in construction, the same bricks can be arranged into different structures, with overall architecture determining function.

Moreover, phenotypic consequences of karyotype alterations often depend on environmental context. Under stable conditions, many karyotype changes may appear phenotypically neutral; however, when environmental constraints are drastically altered, these same karyotypes can produce pronounced and divergent phenotypes. Notably, a similar phenomenon is well documented at the gene level, where many gene mutations do not result in observable phenotypic changes.

Such observations reflect a multi-scale, context-dependent, and inherently “fuzzy” mode of inheritance [76]. Understanding karyotype coding within this framework helps reconcile why certain karyotype changes do not exhibit constant phenotypes while remaining biologically and evolutionarily significant.

## 6. Karyotypes as the System-Level Framework That Provides Informational Context for Inheritance and Biological Organization

Traditional genetics primarily focuses on how specific genes encode heritable traits under defined conditions, implicitly treating genes as independent causal units. In contrast, Genome Architecture Theory (GAT) emphasizes evolution as it occurs in reality, where genetic components (genes and pathways), the entire biological carrier (the organism or cell), and the surrounding environment are inseparable during evolutionary selection. Selection does not act on isolated genes but on integrated systems operating within specific ecological and physiological contexts.

At this larger organizational scale, the system not only constrains and organizes its lower-level components but also serves as an informational environment—or context—for them. Within this context, the same genetic information can acquire different functional meanings or even give rise to novel functions. For example, identical genes can produce distinct phenotypes when placed in different karyotypic configurations, developmental programs, or cellular environments. Similarly, in cancer evolution, the same set of gene mutations can lead to radically different outcomes depending on the karyotype and genomic organization of the cell.

These observations illustrate that higher-level genome architecture does not merely transmit information; it actively shapes, interprets, and transforms lower-level genetic information. In this sense, system-level organization functions as a generator and modulator of biological meaning, enabling the emergence of new traits, adaptive strategies, and evolutionary pathways beyond gene-centric explanations.

For example, a chromosome functions as the immediate environment of a gene, and the same gene can exhibit different functions when placed in different chromosomal contexts. We have previously demonstrated that the chromosomal position of transgenic DNA insertions alters chromatin loop size and gene expression status [87,88,89], a phenomenon widely recognized as the position effect. Extending this concept, we further propose that the same gene may acquire different functional roles when its topological location changes, such as when it is displaced from its original syntenic context.

Similar context-dependent effects apply to epigenetic regulation and other layers of biological coding. For instance, changes in karyotype are expected to alter cellular fitness, which in turn can trigger compensatory adjustments at multiple levels, including gene regulation, organismal behavior, and environmental interactions.

It is also well established that both germline variation and somatic mosaicism contribute to human disease, including cancer and neurodegenerative diseases [90]. Such genomic heterogeneity at the population level can profoundly influence emergent system behavior. As illustrated in cancer evolution, the cellular stress response differs dramatically depending on whether the genomic system is stable or unstable. At different evolutionary stages, either dynamic variation or stabilizing constraints may dominate system behavior.

Taken together, these observations indicate that biological outcomes cannot be fully understood by examining isolated genes or cells alone. Instead, both the karyotype of individual cells and the distribution of karyotypic variants across populations must be considered to synthesize lower-level interactions into coherent system-level behavior.

## 7. Comprehensive Karyotype Profiling Will Play an Increasingly Important Role in Future Biology and Medicine

Two new realizations need more attention. First, genetic determinism, in which using chemical specificity to explain gene specificity seems hugely exaggerated, as biological systems are dominated by fuzziness and relativities. Second, rather than gene-defined molecular specificity, in biological reality, the specificity often can be achieved by a combinational, not very high individual specificity, but by the package level of time, topology, and multiple factors, which, when in isolation, display limited specificity but, within the context of time and topology, contribute good enough specificity. Such specificity is an emergent property and cannot be mapped back to specific components [4,11,13,91,92,93]. This key feature also makes biology unique. The karyotype defines the system-level combinational relative specificity of inheritance, and the process becomes significantly important. Interestingly, such fuzziness and relativity can also be detected at the gene level.

Genome Architecture Theory (GAT) proposes a fundamentally new relationship among energy, matter, and information in biological systems [4,94,95,96,97,98]. In this framework, information plays the primary organizing role: organisms are defined by system-level information, which directs the use of energy to assemble and maintain specific carriers composed of matter—namely, unique biological entities with distinct identities. Energy does not determine organization; rather, it is recruited and constrained by biological information to construct and sustain ordered material structures.

Evolution, in turn, generates not only new biological forms but also new platforms for energy utilization, progressing from simple to increasingly complex metabolic and organizational systems. As informational complexity increases, matter is organized into more elaborate biological carriers, and multiple layers of biological coding emerge, extending beyond genetic sequences to include genome architecture, epigenetic regulation, system-level constraints, and cognitions. Through the dynamic interaction of information, energy, and matter, life becomes possible and progressively more complex and diverse.

Such a relationship has no direct counterpart in the purely physical world, where energy flow can generate transient order but cannot produce heritable informational systems capable of creating new information and directing energy use to construct increasingly complex material carriers. The cyclical interplay among information, energy, and matter is therefore central to understanding biology and evolution: although all three are mutually dependent, increasing informational complexity acts as the primary driver. This perspective underscores the need to prioritize system-level information.

When combined with the concept of biological emergence from combinatorial interactions among components, it is reasonable to propose that karyotype profiles integrate lower-level information, including copy number variations (CNVs), key gene mutations, environment-specific epigenetic regulation, and degrees of heterogeneity spanning molecular, cellular, and population levels. In this sense, the karyotype platform is not replaced by lower-level data but is instead supported and enriched by information across multiple scales. From this perspective, disease can be viewed as a phenotypic manifestation of disordered information management [99].

## 8. Advancing Genomics and Systems Medicine Through Clinical Cytogenetics and Cytogenomics

Clinical cytogenetic research plays a pivotal role in advancing the field of cytogenetics due to its access to large sample sizes, diverse patient populations, multidisciplinary research teams, and the participation of biotech companies developing automated platforms for molecular cytogenetic and cytogenetic analyses. This environment drives technological innovation, including the integration of multiple platforms, high-resolution karyotyping, and AI-assisted analyses. With the introduction of system-level concepts of inheritance—such as karyotype coding and mechanisms of information creation and preservation—clinical cytogenetics becomes essential for understanding the complexity, dynamics, and heterogeneity of biological systems.

In particular, clinical cytogenetic studies provide unparalleled insights into the dynamic processes of cancer evolution, revealing how chromosomal changes correlate with disease progression, treatment response, and patient outcomes. These studies also contribute to systems medicine by linking karyotype-level information to functional consequences, facilitating the interpretation of genomic instability, gene-level alterations, epigenetic regulation, and multi-scale interactions within cells and populations. Furthermore, cytogenomic datasets serve as a rich resource for validating emerging theories of system-level inheritance, integrating genomic and non-genomic contributions to disease across multiple levels, testing predictive models, and refining precision medicine approaches.

Overall, clinical cytogenetics and cytogenomics are not only diagnostic tools—they are drivers of discovery, enabling the integration of molecular, cellular, and system-level information and providing the foundation for a more comprehensive, predictive, and personalized approach to medicine.

## 9. Advances and Implications of Cytogenetic and Cytogenomic Concepts and Techniques

Cytogenetics and cytogenomics offer unique advantages in modern biology and medicine by integrating morphological and genomic features and enabling simultaneous profiling at both individual and population levels, capturing not only population averages but also biologically meaningful outliers. When combined with studies of system stability and instability during somatic evolution, these approaches open many new frontiers in both research and clinical applications. Below, we highlight key areas of application and emerging directions (see Table 1).

- Characterizing previously underreported cytogenetic alterations

Conventional clinical cytogenetic analyses—such as standard karyotype profiling—are largely grounded in frameworks established between the 1960s and 1990s and have traditionally focused on recurrent, stable, or clonal chromosomal alterations. As a result, many cytogenetic abnormalities with highly altered morphology, low frequency, or apparent instability have been difficult to classify and were often dismissed as biologically insignificant, under the assumption that they would be eliminated during evolution or cell selection.

However, emerging evidence indicates that karyotype organization encodes system-level information and that rare or non-clonal alterations—often regarded as “outliers”—can play a decisive role in macroevolutionary transitions. In this context, such atypical cytogenetic structures can no longer be ignored [100]. Instead, they should be systematically documented and characterized, including aberrant mitotic figures, fragmented chromosomes/nuclei, giant nuclei, micronuclear clusters, polyploid or chaotic genomes, and other unconventional configurations [101].

Beyond cataloging these alterations, future studies should focus on elucidating the relationships among different classes of karyotypic abnormalities and their evolutionary trajectories. Establishing baseline frequencies of chaotic or unstable genomes in normal aging populations, as well as their induction under specific stresses such as drug treatments, will be essential. Such efforts will provide critical insights into genome instability, system-level inheritance, and susceptibility to disease, thereby expanding the conceptual and practical scope of clinical cytogenetics.

b. Standardization of NCCA and CCA scoring

Establishing robust and standardized criteria for scoring non-clonal chromosomal aberrations (NCCAs) and clonal chromosomal aberrations (CCAs), as well as for assessing their dynamic interplay, is critical for understanding somatic evolution and for linking chromosomal instability to disease progression and therapeutic response. Historically, clinical cytogenetics has emphasized CCAs, while NCCAs were often underrecognized or excluded from routine reporting [4,101].

Recently, however, there has been growing interest in using NCCAs to address clinically relevant questions. Examples include studies characterizing chemotherapy- or radiotherapy-induced genome chaos—an extreme form of NCCAs—in Hodgkin’s lymphoma patients using multicolor FISH (M-FISH), as well as reports demonstrating that early and widespread chromosomal abnormalities precede detectable cancer development in Fanconi anemia. These findings highlight the clinical value of NCCAs as indicators of genome instability and early disease evolution [102,103].

An urgent challenge for the field is to formally recognize the biological and clinical significance of NCCAs and to incorporate their assessment into laboratory guidelines and reporting standards, following appropriate validation. Standardized NCCA scoring would provide a powerful tool for monitoring genome instability, predicting disease progression, and evaluating treatment-induced evolutionary dynamics, thereby strengthening the role of cytogenetics in systems medicine.

c. Studying phase transitions in evolution and development

Cytogenetics provides a powerful framework for investigating phase transitions between macro- and micro-evolutionary processes during development and the evolution process, including disease progression and therapeutic response. Notably, genome chaos has been observed during early development without resulting in overt abnormal phenotypes, indicating that extensive genomic instability can be tolerated under certain biological contexts. In recent years, increasing evidence has supported this conclusion.

For example, it has been shown that the first mitotic division in human embryos is highly error-prone; nevertheless, these early mitotic errors can be tolerated and contribute to preimplantation mosaicism rather than earlier developmental failure [104]. More recently, live-imaging studies have revealed de novo mitotic errors at the blastocyst stage—including multipolar divisions, lagging chromosomes, and chromosome misalignment—demonstrating that embryonic cells can withstand substantial mitotic instability during early development [105]. Together, these unexpected observations support the existence of “chaotic” genomic behavior during early development and suggest that evolutionary selection may play a critical role in correcting or filtering abnormal genomes over time.

These findings also challenge how clinical cytogenetic diagnostics are applied in reproductive medicine. There is an urgent need to redefine what constitutes a “normal baseline” of karyotypic variation, taking into account developmental stage, individual variability, and dynamic evolutionary context. Similar cytogenetic analyses can also inform both short- and long-term consequences of therapeutic interventions, including treatment safety and evolutionary responses. Ultimately, quantitative assessment of NCCAs should be further developed into clinically useful biomarkers for genome instability and system-level evolutionary dynamics.

d. Investigating somatic genomic mosaicism

Somatic genomic mosaicism is now recognized as a common biological phenomenon that reflects fuzzy inheritance at the system level. Although low-level mosaicism, particularly non-clonal forms, has long been overlooked in clinical and research settings, accumulating evidence indicates that such variation is widespread in normal tissues, aging cellular populations, and disease states. Importantly, somatic mosaicism is not limited to gene mutations but encompasses changes at multiple genomic scales, including copy number variation, epigenetic modification, mitochondrial alterations, and karyotype alteration.

These mosaic genomic states can significantly influence gene expression patterns, epigenetic landscapes, and the integrity of genome architecture. Studying somatic mosaicism therefore provides critical insight into how intra-individual genomic heterogeneity contributes to system-level instability, altered cellular behavior, and disease susceptibility. From an evolutionary perspective, mosaicism generates diverse cellular populations within the same organism, creating a substrate for selection under changing environmental or therapeutic pressures.

Systematic and quantitative comparisons are needed to determine whether the degree and pattern of somatic mosaicism are tissue-specific, age-dependent, or linked to an individual’s inherent genome stability. Equally important is evaluating whether elevated mosaicism predicts differential treatment responses, therapy resistance, or adverse outcomes—particularly in diseases such as cancer, neurodegeneration, and developmental disorders. Integrating cytogenetic profiling of somatic mosaicism into clinical studies will therefore be essential for advancing systems medicine and for understanding how genome instability shapes biological outcomes across scales.

e. The importance of profiling outliers for evolutionary innovation

Traditionally, biological analyses focus on average profiles, thereby disregarding outliers. Statistical models are routinely used to identify and eliminate outliers as noise. However, while most outliers may play minor roles in microevolution—where average behavior dominates—some outliers can act as key drivers of macroevolution, in which the existing population average is replaced by the expansion of these rare variants.

By tracing the transition between micro- and macroevolution, the relationship between outliers and the emergence of a new population average can be systematically studied. Notably, in treatment-induced rapid drug resistance, genome-chaos–generated outliers are often the critical source of resistance. When investigating cellular macroevolution, karyotype analysis therefore plays a vital role, as it captures system-level genomic reorganization rather than gene-level variation [106].

Clearly, new strategies for data collection and analysis that monitor both population averages and outlier profiles are essential for future cytogenetic and evolutionary research.

f. Environmental and other complex diseases

Cytogenetic approaches provide a powerful framework for elucidating how diverse environmental exposures contribute to complex diseases. Conditions such as Gulf War Illness (GWI) and other multifactorial disorders are characterized by heterogeneous clinical phenotypes, in which genomic instability may arise from multiple biological and environmental triggers rather than a single causative factor.

Traditional medical models are largely based on the assumption that the same disease is caused by the same etiological factors and shares a common pathogenic mechanism, leading to standardized diagnostic and therapeutic strategies. Under this framework, GWI did not readily qualify as a defined disease entity because affected individuals presented with highly variable symptoms and lacked a single, unifying cause or mechanism. This view began to change as accumulating evidence showed increased genomic instability in many GWI patients, often accompanied by mitochondrial dysfunction, immune dysregulation, and abnormal stress-response pathways linked to environmental exposures.

We propose that the development of GWI can be conceptualized as a multistage process:

(1) Initial damage stage—exposure to diverse and intense stresses during the Gulf War causes widespread cellular damage;
(2) Cellular evolution stage—while many individuals recover from the initial damage, in others the genome becomes destabilized, triggering ongoing cellular and genomic reorganization;
(3) Illness stage—the altered genome affects multiple cellular systems, resulting in diverse and persistent clinical manifestations [107].

If this staged model is applicable to other environmentally associated or complex diseases, karyotype-based analyses will be essential for monitoring disease progression and guiding management, as they capture system-level genomic alterations rather than isolated gene changes.

Within the framework of Genome Architecture Theory (GAT), information, energy, and matter are integrated, and information mismanagement is proposed as a shared phenotype across many diseases [4]. Because these three components are dynamically interconnected, karyotype-coded system information can directly or indirectly influence both information flow and energy metabolism. Accordingly, new analytical platforms are needed to simultaneously monitor genomic architecture, information flow, and energy dynamics in order to better understand and manage complex disease states.

g. Monitoring genome manipulation

With the maturation of gene therapy and genome-manipulation technologies, artificial gene and genome alterations are expected to become increasingly routine in both research and clinical practice. One of the key challenges for modern cytogenetics is to monitor these interventions and ensure that engineered changes do not compromise genome stability or system-level integrity.

Comparative studies of commonly used molecular manipulation methods indicate that many can induce varying degrees of genomic instability, particularly at the chromosomal and karyotype levels. Of particular concern, CRISPR/Cas9-based platforms—while highly efficient—can generate unintended genomic alterations, including off-target mutations, structural variants, and chromosomal rearrangements (deletion, amplification, translocation, and aneuploidy) that are not readily detected by sequence-based assays alone [108,109,110,111].

High-resolution cytogenetic approaches are therefore essential for evaluating the full consequences of genome editing. These methods enable the detection of large-scale structural changes, genome reorganization, and karyotype instability, providing critical information that complements molecular analyses. Such system-level monitoring is indispensable for assessing the safety, reliability, and long-term stability of engineered cells intended for both experimental and therapeutic applications.

h. Integrative combinatorial platforms

Over the past two decades, the integration of karyotype profiling with fluorescence in situ hybridization (FISH), microarray technologies, and sequencing has become routine. However, these integrations have largely been guided by a gene-centric framework, in which chromosomal information is primarily interpreted as a scaffold for gene-level variation. As a result, system-level genome organization and instability have often been underappreciated.

A new integrative paradigm is required, one that is grounded in Genome Architecture Theory and focuses on karyotype coding and overall system instability as primary biological information. This shift demands analytical frameworks that treat genome architecture as an active carrier of inheritance and evolutionary potential, rather than as a passive structure.

From a technological and data-integration perspective, advanced bioinformatic tools are needed to translate information across scales—from sequence-level data to karyotype-level organization [112,113]. By combining cytogenetic data with gene-level and epigenetic information, truly multiscale analyses of system-level inheritance become possible. Such integrative platforms can reveal how genome architecture, epigenetic regulation, and environmental inputs collectively shape cellular behavior and evolutionary trajectories.

At the same time, effective synthesis of data from diverse genome-scale platforms is essential, including automated and high-resolution technologies such as fiber-FISH and optical genome mapping [114,115,116]. The development of combinatorial platforms that unify these approaches will be critical for advancing cytogenetics from descriptive analysis to predictive, system-level genome monitoring. Moreover, many current molecular platforms need to be combined with in situ features so that morphological and topological information can be integrated. Some of the frequently used cytogenetic and cytogenomic methods are listed in Table 1.

**Table 1:** Examples of cytogenetic and cytogenomic platforms and the rationale for future directions.

A. Principles for designing and performing cytogenetic analyses	The limitations of molecular profiling in general, and NGS in particular, also exist at the conceptual level. The following categories represent different approaches and rationales for using cytogenetic platforms.	
	Gene-centric units vs. karyotype-defined system inheritance	[4,35]
	Technical noise vs. fuzzy inheritance	
	End products vs. dynamic evolutionary process (aging/disease)	
	Average profiling vs. average + outliers	
	Physiological constraints versus pathological uncertainty	
	Fixed pattern vs. pattern + stochasticity	
	Classic aberrations vs. classic aberrations + novel types	
	Microevolutionary constraints vs. macroevolutionary dynamics	
	Precise biological processes vs. chaotic processes under stress	
	Normal cell biology vs. new modes of information creation	
	Specific genetic aberrations vs. overall system-level instability	
	Diagnosis vs. differential diagnosis, plus monitoring and predicting treatment response, including genome manipulation	
B. Examples of current methods	Some of these methods are used primarily in research laboratories and have not yet been adopted in clinical laboratories	
	NGS	[117]
	Single-nucleotide polymorphism array and array comparative genomic hybridization	[38]
	Profiling somatic genomic mosaicism	[90]
	FISH on complex small supernumerary marker chromosomes	[118]
	Multitarget FISH diagnostic applications	[119,120,121,122]
	SKY and M-FISH	[123,124]
	High-resolution fiber–FISH	[114,115]
	Halo–FISH profiling chromatin loops	[87]
	DNA–protein co-detection in situ	[88]
	SKY–protein co-detection in meiosis	[87,89]
	Profiling Chromosome Topological 3D Structure	[125]
	Profiling chromosome territories and loop dynamics	[87,125,126,127,128]
	Studying telomere architecture	[129,130]
	Refining chaotic genomes, including massive translocations	[4,50,52,55,57,58,75,77,78,79,80,101,131]
	chromothripsis, chromoplexy, chromoanagenesis	
	sticking chromosomes, defective mitotic figures, giant nuclei, micronuclei clusters, nuclear eruption	
	chromosome fragmentation, and other drastic chromosomal/nuclear alterations	
	Optical genome mapping	[116]
	Multiplexed error–robust FISH or MERFISH	[132]
	High-throughput DNA FISH (hiFISH)	[133]
	Advanced platforms beyond mutation and expression profiles	[134]
	Tools for converting DNA data into cytogenetic views	[134,135,136,137]
	Web resources for cytogeneticists	[138]
C. Validation, prioritization, and establishment of combinatorial platforms	The various technical platforms need to be systematically compared and validated against one another. At present, different platforms are being used independently by different researchers. To achieve these goals, the following relationships need to be systematically examined using the same model system. Such comparisons can provide a scientific rationale for prioritizing methods according to the questions being addressed, rather than continuing to use a given platform simply because of existing expertise with that method.	
	The independent, additive, synergy, or conflict relationship	[4,10]
	among different platforms.	
	The dynamic relationships among different stages of evolution often	
	involve opposing effects, particularly between	
	macroevolutionary innovation and microevolutionary constraints.	
	The different responses under different levels of stress	
	Identify the disease condition and the primary level of inheritance involved. For example, in cancer diagnosis, because both macro- and microevolutionary processes are involved, genome-level alterations should be prioritized. In other words, cytogenetic methods are fundamentally important.	
D. Future platforms	One of the key tasks for future cytogenetics is to establish an information-management–based evolutionary framework at the system level. Such a framework would enable evaluation of the strengths and limitations of different genetic platforms, with appropriate prioritization of diseases according to disease characteristics, the stage of somatic evolution, and the scale of biological organization involved (from genes to genomes, cells, tissues, individuals, and populations). Different clinical scenarios, therefore, require different combinatorial analytical strategies.	[4,9,10]
	Within this framework, cytogenetics provides an essential informational context for interpreting gene mutations and copy number variations, as it defines the meaning of lower-level variation and genomic dynamics. These lower-level dynamics depend on system-level stability, the degree of environmental stress, and clinical priorities. Currently, genetic analysis is largely centered on individual gene function. In the future, however, analytical emphasis should shift toward the genomic system as a whole, with overall patient benefit as the primary goal, within which gene-level data are integrated and interpreted.	
	Establishment of data-conversion platforms	[4,10,50,138]
	Converting DNA-level datasets into karyotype-level data	
	Interpreting identical DNA datasets by karyotype states	
	Defining prioritization rules when DNA-level information conflicts with karyotype-level organization	
	Providing a platform for simplifying and integrating multi-level genomic profiling	
	Validation and application of karyotype coding	
	Validating the concept of karyotype coding by experiments	
	Integrating key molecular profiles into karyotype-based	
	Analyses like transposable elements and epigenetics	
	Development of combinatorial analytical platforms	
	Establishing combinatorial platforms rather than a single unified model	
	Employing context-dependent sets of sub-models tailored to disease type, evolutionary stage, and system scale	
	Automation of cytogenetic platforms	
	Currently, both the procedures and analyses are less automated than NGS and need improvement (e.g., OGM)	
	AI-assisted cytogenetic platforms	
	Integrates cytogenetic and molecular data across scales	
	Reexamine the relationship between NCCAs and CCAs across a large sample size	
	Applies context-specific combinatorial frameworks to guide platform selection and data interpretation

i. Modeling evolutionary dynamics and addressing fundamental biological questions

Diseases—particularly cancer—serve as powerful natural models of evolutionary processes, as each tumor or patient sample represents an independent evolutionary trajectory. Cytogenetic analyses across large sample sets therefore provide a unique opportunity to examine system behavior, evolutionary constraints, and adaptive mechanisms, offering direct insight into fundamental biological principles.

As discussed in this perspective, the two-phased evolutionary framework emerged in part from studies of cancer evolution. Observations of rapid, discontinuous genome reorganization followed by periods of relative stability challenge the central assumption of strictly gradual change in classical Darwinian evolution and instead highlight the importance of genome-level reorganization in macroevolutionary transitions [4,98].

Beyond evolutionary theory, the extensive diversity of karyotypes observed in disease contexts also provides valuable material for addressing core questions in cell biology, genomics, and systems biology. These include the organization and function of chromatin loops, the role of genome topology as a form of biological information, and the dynamic relationships among energy, matter, and information within living systems.

We anticipate that advances in clinical cytogenetics and cytogenomics will not only improve disease diagnosis and management but will also make substantial contributions to basic biological research by illuminating the system-level principles that govern cellular organization, evolution, and function.

## 10. Conclusions

By revisiting the current landscape of cytogenetics and cytogenomics, we emphasize the enduring and growing conceptual importance of karyotype profiling as a central platform in clinical cytogenetics. Far from being rendered obsolete by sequencing technologies, cytogenetics provides the system-level perspective necessary to understand genome organization, inheritance, heterogeneity, and evolutionary dynamics—dimensions that are essential for interpreting development, disease, and treatment response.

As new concepts such as karyotype coding, system-level inheritance, and information creation and preservation gain traction, clinical cytogenetics is poised to play an increasingly important role in future genomics and systems medicine. Emerging avenues—including high-resolution karyotype profiling, integrative cytogenomic platforms, AI-assisted analysis, and evolutionary modeling of disease—present both significant challenges and unprecedented opportunities for the field. For example, because CIN represents a major driver of cancer evolution with clear clinical relevance [135,136,139,140,141,142], cancer cytogenetics is expected to play an increasingly important role in monitoring CIN for treatment management.

This perspective is intended not only to highlight these new frontiers but also to stimulate discussion and critical reflection within the cytogenetics and cytogenomics communities. One of us, HH, as a molecular cytogeneticist who has benefited profoundly from the concepts, methodologies, and intellectual legacy of cytogenetics, remains committed to advancing these ideas and to continuing this dialogue as opportunities arise. Ultimately, the future of cytogenetics will depend on a new generation of researchers willing to engage with complexity, embrace system-level thinking, and carry forward the task of redefining how we understand genomes, inheritance, and evolution in health and disease.

## Data Availability

No new data were created or analyzed in this study.
